# Semi-Industrial Production of a DPP-IV and ACE Inhibitory Peptide Fraction from Whey Protein Concentrate Hydrolysate by Electrodialysis with Ultrafiltration Membrane

**DOI:** 10.3390/membranes12040409

**Published:** 2022-04-09

**Authors:** Mélanie Faucher, Thibaud R. Geoffroy, Jacinthe Thibodeau, Sami Gaaloul, Laurent Bazinet

**Affiliations:** 1 Institute of Nutrition and Functional Food (INAF) and Food Science Department, Pavillon Paul Comtois, Université Laval, Quebec City, QC G1V 0A6, Canada; melanie.faucher.2@ulaval.ca (M.F.); thibaud.geoffroy.1@ulaval.ca (T.R.G.); jacinthe.thibodeau.1@ulaval.ca (J.T.); 2 Laboratoire de Transformation Alimentaire et Procédés Électro-membranaires (LTAPEM, Laboratory of Food Processing and Electro-Membrane Processes), Pavillon Paul Comtois, Université Laval, Quebec City, QC G1V 0A6, Canada; 3 Lactalis Canada, Victoriaville, QC G6P 9V7, Canada; sami.gaaloul@ca.lactalis.com

**Keywords:** whey protein concentrate, bioactive peptides, electrodialysis with ultrafiltration membrane, antioxidant activity, DPP-IV inhibition activity, ACE inhibition activity

## Abstract

The separation by electrodialysis with ultrafiltration membranes (EDUF), at a semi-industrial scale, of a new whey protein hydrolysate obtained from a whey protein concentrate was assessed. After 6 h of treatment, more than 9 g of peptides were recovered in the peptide recovery fraction, for a recovery yield of 5.46 ± 0.56% and containing 18 major components. Among these components, positively charged peptides, such as ALPMHIR + PHMIR, LIVTQTMK and TKIPAVF, were present, and their relative abundances increased by nearly 1.25 X and up to 7.55 X. The presence of these peptides may be promising, as ALPMHIR has a strong activity against angiotensin-converting enzyme (ACE), and LIVTQTMK has structural properties that could interfere with dipeptidyl peptidase-IV (DPP-IV). Many neutral peptides were also recovered alongside those. Nevertheless, the inhibitory activity against DPP-IV and ACE increased from 2 X and 4 X, respectively, in the peptide recovery fraction compared to the initial hydrolysate, due to the improved content in bioactive peptides. Thus, this new hydrolysate is well-suited for the large-scale production of a peptide fraction with high bioactivities. Furthermore, what was achieved in this work came close to what could be achieved for the industrial production of a bioactive peptide fraction from whey proteins.

## 1. Introduction

Over the last few years, various food proteins have been used as a source of bioactive peptides following their hydrolysis. These peptides have demonstrated promising health effects, making them an interesting avenue to promote health and reduce the risks of some diseases. Among food proteins, whey proteins are attractive, as many bioactive peptides were identified from them [1]. Nevertheless, since many peptides are produced from the hydrolysis of whey proteins, some having no activity, some having antagonist activity, it is interesting to purify or to concentrate together such bioactive peptides to increase their activity [1,2]. Until recently, among the technologies available to do so, pressure-driven membrane processes, such as ultrafiltration (UF) and nanofiltration, have remained the most applicable at a large-scale [3]. However, since they allow the separation of peptides mainly based on the molecular weight, it could be challenging to have a selective process and final fractions with higher activity. Nevertheless, an innovative and ecofriendly technology has lately been developed to selectively recover bioactive peptides.

Electrodialysis with ultrafiltration membrane (EDUF) combines electrodialysis with filtration membranes, making it possible to separate components based on their charge and molecular weight. This technology has many applications [4,5,6], but since its development, ithas mainly been used to separate peptides from various sources [7,8,9], and the parameters governing the process and peptide separation have been widely studied (membrane types, electric field strength, flow rate, pH, etc.) [5,6,10,11,12,13]. EDUF was tested at different scales and recently, for the very first time, its feasibility at a semi-industrial scale was proven [14,15]; a hydrolysate from a whey protein isolate (WPI) (Prolacta, Lactalis, Retiers, France) obtained with a pure enzyme from Sigma-Aldrich was separated with an EUR-6 cell (the smallest industrial cell commercially available). Using an optimal configuration, the selective recovery of bioactive peptides was obtained, such as ALPMHIR and IPAVFK. An increase in the bioactivity of the recovery fraction was observed in comparison with the initial hydrolysate for dipeptidyl peptidase-IV (DPP-IV) inhibition activity and angiotensin-converting enzyme (ACE) inhibition activity of 4 and 6 X, respectively [15]. These results confirm the application of EDUF at a large scale and suggest its possible use to commercially produce bioactive peptides. Therefore, to continue the development of this technology, it remains important to assess the selectivity and yield of the process at a large scale on various hydrolysates of varying complexities, and, particularly, with a product that is easily accessible in the industry and with a commercial enzyme available at the industrial scale.

In this context, a new hydrolysate, made from a common whey protein concentrate (WPC35), was separated by EDUF at a semi-industrial scale. Indeed, the objectives of this work were: (1) to study the impacts of the process on the electrodialytic parameters, (2) to assess the migration of the major bioactive peptides and the selectivity of the process and (3) to evaluate the impact of the process on the bioactivity of the fractions produced.

## 2. Materials and Methods

### 2.1. Whey Protein Hydrolysate

To prepare the initial whey protein concentrate hydrolysate (WPH), a commercial WPC35 provided by Lactalis (Victoriaville, Qc, Canada) was used with a commercial trypsin VI from porcine pancreas containing 11% of chymotrypsin (trypsin activity ≥2400 USP units/mg, chymotrypsin activity ≥400 USP units/mg) (Neova Technologies Inc., Abbotsford, BC, Canada). Prior to hydrolysis, a solution of 3.5% protein was made with the WPC and was left to rehydrate overnight at 4 °C under a moderate agitation. On the day of hydrolysis, the temperature and pH of the WPC solution were adjusted to 37 °C and 8.0, respectively (the pH was adjusted with NaOH), under a constant agitation. Then, the industrial enzyme was added to the solution at a ratio of 1:200 (enzyme:substrate), and the hydrolysis was carried out during four hours while the pH and temperature were continuously maintained. After four hours, the reaction was stopped by inactivating the enzyme with a thermal treatment (80 °C, 30 min). Then, the hydrolysate was cooled at 15 °C, and the pH was adjusted to 7.0 with a HCl solution. The hydrolysate was then frozen in aliquots at −28 °C, and the WPH was thawed before the EDUF experiments.

### 2.2. Isolation of Peptide from the Whey Protein Hydrolysate

#### 2.2.1. Electrodialysis Cell

**Figure 1:**
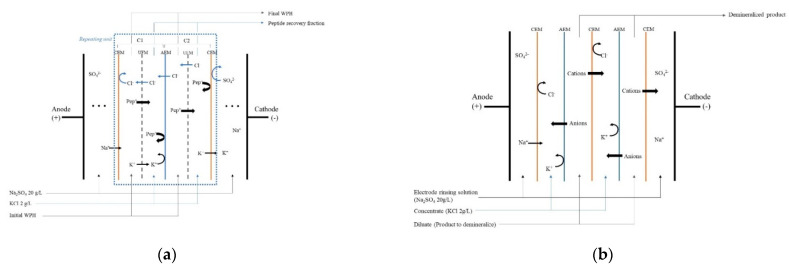
(**a**) Electrodialysis with ultrafiltration membrane cell configuration used to recover cationic peptides from whey protein concentrate hydrolysate; (**b**) electrodialysis cell configuration used to demineralize initial whey protein concentrate hydrolysate, peptide recovery fraction and final whey protein concentrate hydrolysate.

The EDUF experiments were carried out using the smallest industrial unit commercialized by Eurodia Industrie SAS (Pertuis, France), with an effective surface of 560 cm² designed to recover positively charged peptides (Figure 1a). The cell configuration used, developed by Goeffroy et al. [14], was designed to overcome some technological limitations, such as demineralization of the recovery compartment, loss of selectivity, etc., encountered in a previous work. Thereby, as described in detail by Geoffroy et al. [14], the cell was composed of cation-exchange membranes (CEMs) (Astom, Tokyo, Japan), anion-exchange membranes (AEMs) (Astom, Tokyo, Japan) and UF membranes (Synder, Vacaville, CA, USA) stacked between two dimensionally stable electrodes (DSE). The UF membrane was selected from the work of Kadel et al. [16], since the authors demonstrated an interesting positively-charged-selective peptide migration, allowing the maximum recovery of certain peptides of interest, such as peptides having strong bioactivities, while limiting the migration of negatively charged peptides.

All membranes allowed the formation of compartments where the WPH, the peptide recovery fraction and the electrode rinsing solution could circulate. Furthermore, the EDUF cell was composed of repeating units, themselves composed of two different cells (C1 and C2) (Figure 1a). Both compartments allowed the positively charged peptides to migrate from the WPH to the peptide recovery fraction through a UF membrane. However, in C1, K^+^ and Cl^−^ ions were kept in the peptide recovery fraction and the WPH, while they could migrate in C2. Therefore, the ion concentrations were theoretically maintained during treatment. Furthermore, during treatment, it was possible for the solutions to circulate due to closed loops, reservoirs and pumps.

#### 2.2.2. Protocol

A 20 g/L Na_2_SO_4_ solution was used as the electrode rinsing solution, whereas a 2 g/L KCl solution was used for the peptide recovery fraction. During EDUF treatment, 4 L of each solution was treated (WPH, electrode rinsing solution and peptide recovery fraction), and to limit microbiological growth, their temperature was maintained between 17–19 °C with a cooling system.

In total, 4 EDUF trials were performed, and each treatment was carried out for 6 h under a constant current, as in industrial electrodialysis applications. Here, a current of 1.2 Amperes was chosen according to previous works [14,15]. The pH of the WPH was also kept constant at 7.0 using NaOH and HCl solutions. During treatment, 2 mL samples of the WPH and peptide recovery fraction were taken at t = 0, 0.5, 1, 2, 3, 4, 5 and 6 h to perform global peptide migration quantification (BCA) and the individual peptide migration (LC-UV). These samples were frozen at −28 °C and thawed at 4 °C before being used. Furthermore, the conductivity of the WPH and peptide recovery fraction, as well as the current intensity and voltage were measured every 10 min for the first hour of the assay and every 20 min for the remaining time. After each treatment, the EDUF cell was washed following the manufacturer’s practices.

After each EDUF assay, the peptide recovery fraction and the final WPH were recovered and freeze-dried. The freeze-dried products of all assays were pooled together before being demineralized or used for analyses.

### 2.3. Demineralization of Initial WPH, Final WPH and Peptide Recovery Fraction

#### 2.3.1. Electrodialysis Cell

The pooled initial WPH, final WPH and peptide recovery fraction were demineralized using a MP type cell (ElectroCell AB, Täby, Sweden), and the cell configuration was made of alternating CEMs and AEMs (Astom, Tokyo, Japan) (Figure 1b), allowing the formation of compartments for the electrode rinsing solution, concentrate and diluate. The anode was a dimensionally stable electrode (DSA-O_2_), and the cathode was a food-grade stainless steel electrode. During treatment, all solutions were circulating through three closed loops connected to reservoirs, pumps (Baldor Electric Company, Fort Smith, AR, USA) and flowmeters (Blue-White Industries Ltd., San Diego, CA, USA).

#### 2.3.2. Protocol

To perform the demineralization treatment, the freeze-dried and pooled samples (initial WPH, final WPH and peptide recovery fraction) were solubilized in deionized water before being demineralized up to 90% (using the conductivity values). Thus, the treatment time was consequently adjusted. The demineralization treatments were performed under a constant voltage of 9 V. The electrode rinsing solution was a 20 g/L Na_2_SO_4_ solution (800 mL), and the concentrate solution was a KCl 2 g/L solution (500 mL). Moreover, the flow rates were 500 mL/min for the diluate and concentrate and 1000 mL/min for the electrode rinsing solution.

After demineralization, the demineralized initial WPH, demineralized peptide recovery fraction and demineralized final WPH were recovered and freeze-dried.

### 2.4. Analyses

#### 2.4.1. Conductivity and Local Electric Field Strength

The conductivity of the WPH and peptide recovery fraction (for EDUF treatments) and the concentrate and diluate (for demineralization treatments) was measured using an YSI conductivity meter (Model 3100, Yellow Springs Instrument, Yellow Spring, OH, USA) equipped with an YSI immersion probe (Model 3252, cell constant K = 1 cm^−1^).

For EDUF treatments, the local electric field strength of the WPH (in V/cm) was calculated as [17]:

(1) $$E_{f}=\frac{I}{\sigma_{\mathrm{WPH}}\times A}$$

where I is the current intensity (in Amperes), directly obtained on the power supply; σ_WPH_ is the conductivity of the WPH (in S/cm); and A is the membrane area (in cm^2^).

#### 2.4.2. Peptide Recovery, Yield and Migration

The global peptide concentration in the peptide recovery fraction during EDUF treatment was determined using a BCA protein assay (Pierce, Rockford, IL, USA) following the manufacturer’s instructions. To perform the analysis, the liquid samples, that were collected during EDUF treatment, were used (Section 2.2.2). Briefly, 25 µL of sample was mixed with 200 µL of working reagent in a microplate. The microplate was incubated at 37 °C for 30 min, and then cooled at room temperature for 10 min, before being read at 562 nm with a microplate reader (x-Mark, Bio-Rad, Hercules, CA, USA). The peptide concentration in the sample was determined with a calibration curve ranging from 0 to 1000 µg/mL of bovine serum albumin (BSA).

Using the initial peptide concentration during EDUF treatment and the volume treated (including the dead volume of the EDUF system), the mass of peptides recovered (g) in the peptide recovery fraction during treatment was calculated. Knowing the mass of the peptide, it was possible to determine the peptide migration rate (in g/h) as:

(2) $$\mathrm{Peptide}\;\mathrm{migration}\;\mathrm{rate}=\frac{M_{\mathrm{peptides}}}{t}$$

where M_peptides_ is the mass of the peptide recovered in the peptide recovery fraction (g), and t is the duration of the treatment (h).

Furthermore, using the peptide concentration of the peptide recovery fraction during EDUF treatments and knowing the initial peptide concentration of the WPH, it was possible to calculate the peptide yield (%) during treatment, such as:

(3) $$\mathrm{Peptide}\;\mathrm{yield}=\frac{C_{\mathrm{peptides}}}{C_{\mathrm{initial}}}\;\times \;100$$

where c_peptides_ is the peptide concentration of the peptide recovery fraction (in g BSA eq/mL), and c_initial_ is the peptide concentration in the initial WPH (g BSA eq/mL).

#### 2.4.3. Individual Peptide Migration

The liquid samples collected during the EDUF treatment were used to analyze the individual peptide migration during treatment and their final migration. Before their injection into the LC-UV-MS/MS system, the samples were filtered with a 0.22 mm PVDF filter. The equipment and operating parameters were the same used by Geoffroy et al. [15]. The UV detection data allowed the identification of the major components in the peptide recovery fraction, and their area under the curve (AUC) was used for semi-quantification. The identification of the individual peptide sequences was carried out using the MS/MS data and software, the UniProt database and the FindPep tool.

Knowing the AUC of each individual component, it was possible to follow the migration kinetic of each peptide throughout EDUF treatment. Moreover, the final migration rate of each peptide was calculated as the ratio between the AUC in the peptide recovery fraction and the AUC in the initial WPH.

#### 2.4.4. Total Peptide Content

The nitrogen content of the freeze-dried samples (initial WPH, peptide recovery fraction, final WPH, demineralized initial WPH, demineralized peptide recovery fraction and demineralized final WPH) was measured by the Dumas combustion method using a Rapid Micro N Cube (Elementar Analysensysteme GmbH, Langenselbold, Germany). The peptide content was calculated with 6.38 as the conversion factor from nitrogen to protein [18].

#### 2.4.5. Peptide Relative Abundance

The relative abundance of the major peptides previously identified (Section 2.4.3) was determined in the initial WPH, peptide recovery fraction, final WPH, demineralized initial WPH, demineralized peptide recovery fraction and demineralized final WPH. To do so, for each sample, a solution containing 0.5% (*w*/*v*) peptide was prepared, before being filtered (0.22 μm PVDF filter) and analyzed. The same equipment and operating parameters as the ones previously described were also used here (Section 2.4.3). The relative abundance was determined as the ratio between the AUC of each component and the AUC of all components for a sample [16].

#### 2.4.6. Bioactivity Assay

- DPP-IV inhibitory activity

The colorimetric assay from the Enzo DPP-IV Drug Discovery Kit (Enzo Life Sciences, Farmingdale, NY, USA) was used to measure the DPP-IV inhibitory activity, an indicator of the potential antidiabetic effects of the demineralized initial WPH, demineralized final WPH and demineralized peptide recovery fraction [19]. Prior to analysis, the dry samples were solubilized in the assay buffer (50 mM Tris, pH 7.5) at a peptide concentration of 2.86 mg/mL following preliminary tests. Briefly, 15 µL of DPP-IV enzyme (35 mU solution diluted 50 times in buffer) and 35 µL of the prepared sample were mixed in a microplate. A positive and a negative control were obtained by replacing the sample with a 10 µL P32/98 inhibitor (100 µM) with 25 µL of assay buffer and 35 µL of buffer, respectively. Moreover, a blank was prepared by adding only 50 µL of assay buffer in the wells. The plate was incubated at 37 °C for 10 min, before 50 µL of substrate (H-Gly-Pro-*p*-nitroaniline, 200 µM) was added. The absorbance was then read at 405 nm every minute for 30 min (xMark microplate absorbance spectrophotometer, Bio-Rad, Hercules, CA, USA). Once the slope of the function was obtained, the percentage of inhibition (%) was calculated as suggested by the manufacturer:

(4) $$\mathrm{Percentage}\;\mathrm{of}\;\mathrm{inhibition}=\left(1-\frac{{\mathrm{slope}}_{\mathrm{Absorbance}\;\mathrm{sample}}}{{\mathrm{slope}}_{\mathrm{Absorbance}\;\mathrm{negative}\;\mathrm{control}}}\right)\times 100$$

For all samples, the half-maximal inhibitory concentration (IC_50_) values were calculated and were expressed in mg of peptide/mL.

b. ACE inhibitory activity

The ACE inhibitory activity of the demineralized initial WPH, demineralized final WPH and demineralized peptide recovery fraction was analyzed using a spectrophotometric method adapted from [20,21] to provide an overview of the antihypertensive potential of the samples. Briefly, the dry demineralized samples were solubilized in deionized water at a peptide concentration of 5 mg/mL following preliminary tests. To perform the analysis, 20 µL of the solubilized sample (or 20 µL of either deionized water or Enalapril, acting as a blank or a positive control, respectively) was mixed with 20 µL of ACE enzyme (0.25 U/mL prepare in borate buffer) and 80 µL of pH 8.3 borate buffer in Eppendorf tubes, before being vortexed for 30 s and incubated at 37 °C for 10 min. For each sample (demineralized initial WPH, demineralized final WPH and demineralized peptide recovery fraction), a negative control was also prepared by mixing the solubilized sample, enzyme and buffer and boiling at 95 °C for 10 min. After cooling down on ice, 40 µL of 6.25 mM of ACE substrate N-hippuryl-His-Leu (HHL) was added in all tubes, and they were vortexed for 30 s before being incubated at 37 °C for 1 h. Then, the tubes were heated to 95 °C for 10 min. After heating, 480 µL of borate buffer and 360 µL of 2,4,6-trichloro-s-triazine (TT) were added, and the tubes were vortexed for 30 s. The tubes were then centrifuged, and 200 µL of supernatant was put into a well of a 96-well clear microplate. The absorbance of the samples, controls and blanks was then read with a spectrophotometer (xMark microplate absorbance spectrophotometer, Bio-Rad, Hercules, CA, USA) at 382 nm. The percentage of inhibition (%) was calculated as [14]:

(5) $$\mathrm{Percentage}\;\mathrm{of}\;\mathrm{inhibition}=\left(\frac{{\mathrm{Absorbance}}_{\mathrm{negative}\;\mathrm{control}}-{\mathrm{Absorbance}}_{\mathrm{sample}}}{{\mathrm{Absorbance}}_{\mathrm{negative}\;\mathrm{control}}-{\mathrm{Absorbance}}_{\mathrm{blank}}}\right)\times 100$$

For all samples, the IC_50_ values were calculated in mg of peptide/mL.

c. Antioxidant activity

The antioxidant activity of the demineralized initial WPH, demineralized final WPH and demineralized peptide recovery fraction was determined using the oxygen radical absorbance capacity (ORAC) method [21,22]. Briefly, the dry samples were solubilized in a 75 mM pH 7.4 phosphate-buffered solution at a protein concentration varying from 0.0391 mg/mL to 0.5 mg/mL. Trolox standards (12.5–25–50–100 µM) were also prepared (in the buffer solution). The samples were mixed with 150 µL of 0.1 mM fluorescein in a black 96-well microplate. The plate was incubated at 37 °C for 30 min in the dark. Then, 50 µL of 150 mM 2,2′-azobis(2-methylpropionamidine) dihydrochlorine (AAPH) solution was added. The fluorescence was read for 90 min, every minute, at an excitation wavelength of 485 nm and an emission wavelength of 538 nm (Spectrophotometer Synergy H1, Biotek, Winooski, VT, USA). The results were expressed as micromoles of Trolox equivalent per gram of peptide (mg Trolox eq/g of peptide), as allowed by the Trolox calibration curve [14].

#### 2.4.7. Lactose Content

The freeze-dried initial WPH, peptide recovery fraction and final WPH lactose content was analyzed by high-performance liquid chromatography (HPLC) [23]. Briefly, to perform the analysis, 0.2 g of the sample was solubilized in HPLC-grade water before being treated with Biggs–Szijarto solution. The solution was then centrifuged (5000× *g*, 5 min, 10 °C). The supernatant was recovered and diluted 10X in HPLC-grade water before being filtered with a 0.45 mm nylon filter. For the HPLC measurements, an Agilent 1100 Series chromatograph (Santa Clara, CA, USA) equipped with an Agilent 1260 Infinity refractive index detector (Santa Clara, CA, USA), a column oven and a cooled 717 Plus autosampler were used. The previously prepared samples were injected onto an ICSep-ICE-ION-300 column (Transgenomic, Omaha, NE, USA), and the mobile phase was a solution of H_2_SO_4_ (180 μL/L) at a flow rate of 0.4 mL/min. The column was kept under a constant temperature of 40 °C, and the run time was 45 min. To perform the quantification, a standard of lactose anhydrous (Sigma Company, Saint-Louis, MO, USA) was used.

### 2.5. Statistical Analysis

The data obtained were reported as mean value ± standard deviation, and they were subjected to one-way analyses of variance (ANOVA) to compare the bioactivities of the fractions produced. Statistical differences between the fractions were analyzed by Tukey test (*p* < 0.05) using SigmaPlot software (version 12, Systat Software, San Jose, CA, USA).

## 3. Results and Discussion

### 3.1. Process Parameters

#### 3.1.1. Conductivity of the Solutions

**Figure 2:**
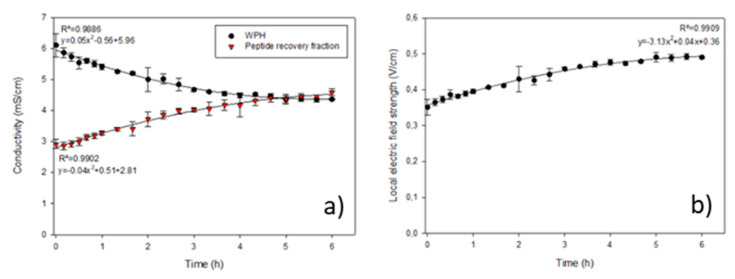
Evolution as a function of time of (**a**) the conductivity of the whey protein hydrolysate (WPH) and peptide recovery fraction and (**b**) the local electric field strength in the WPH.

The evolution in the conductivity of the WPH and peptide recovery solution during EDUF treatment is presented in Figure 2a. During EDUF treatment, the conductivity of the WPH decreased (loss of conductivity of 1.74 ± 0.37 mS/cm), while the conductivity of the peptide recovery fraction increased by 1.67 ± 0.20 mS/cm. Thus, the conductivity of both compartments seemed to reach an equilibrium around 4.5 mS/cm after more than 4 h of treatment. However, prolonging the treatment to see whether these values would be stable and maintained over time would be necessary. Nevertheless, the change in the conductivity of the solutions is due to ion migration, allowed by the cell configuration (Figure 1a), although it was optimized to minimize it.

Such a conductivity loss in the WPH during treatment was previously reported for the same cell configuration, but with a different hydrolysate. Indeed, it is important to control the conductivity since its evolution during treatment can impact the local electrical field strength, which is an important parameter during EDUF treatments [11,14].

#### 3.1.2. Local Electric Field Strength

During EDUF, the local electric field strength of the WPH slightly increased to reach a plateau (Figure 2b), following the inverse of the tendency observed for the change in conductivity. Such an increase in the local electric field strength was already reported for the same type of EDUF cell, but with a different WPH (4.0%) [15]. However, due to the nature of the WPH and its initial conductivity, the values of the electric field strength reached here (around 0.35 to 0.50 V/cm) are lower than the ones previously reported (around 0.50 to 0.75 V/cm).

Nevertheless, as the local electric strength changed during EDUF treatment, it would be beneficial to keep it constant at slightly higher values. Indeed, it was previously demonstrated that a relatively constant local electric field strength in the WPH compartment could positively affect peptide migration, particularly on process selectivity [14], as the migration of peptides of interest may increase.

### 3.2. Peptide Recovery, Yield and Migration Rate

**Figure 3:**
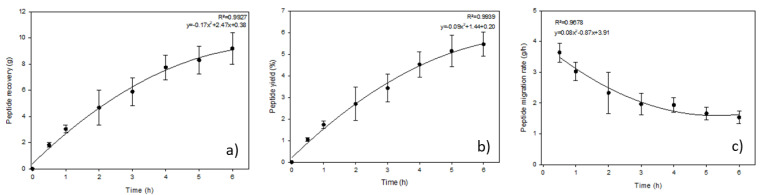
Evolution as a function of time of (**a**) peptide recovery, (**b**) peptide yield and (**c**) peptide migration rate in the peptide recovery fraction.

The peptide recovery, yield and migration rate in the peptide recovery fraction are presented in Figure 3. Not surprisingly, both the peptide recovery and yield increased during the EDUF process, as peptides migrated from the WPH to the peptide recovery compartment. Thereby, after 6 h of treatment, 9.19 ± 1.19 g of peptides were recovered, corresponding to a yield of 5.46 ± 0.56%. Nevertheless, the peptide migration rate decreased during the process, as the rate passed from 3.64 ± 0.31 to 1.53 ± 0.20 g/h, tending towards a plateau after 3 h of treatment (Figure 3c). Two phenomena could explain this reduction in the peptide migration rate: (1) organic fouling on the UF membranes, although the UF membranes in the cell are among the membranes least prone to fouling [24,25]; (2) a decrease in the availability of peptides being able to easily migrate due to their charge and size in the WPH [14]. In addition, after three and a half hours of treatment, there is a change in the shape of the slope for the peptide recovery and yield curves, as the peptide migration rate reaches a plateau.

It is also important to mention that the peptide recovery, yield and migration rate achieved in these experiments are higher than the ones reached for another WPH prepared at 4% and separated with the same EDUF cell [15]. Indeed, for the same treatment time, 2.79 additional grams of peptides were recovered corresponding to an increase in the peptide yield of 0.96 unit of percentage. Furthermore, the migration rate was 1.39 to 1.46 X higher than previously reported. Thus, the results obtained are promising in terms of global peptide recovery. However, it remains important to verify that the recovery of the peptides is selective and consistent with the chosen cell configuration, as well as the final bioactivities obtained.

### 3.3. Peptide Identification and Semi-Quantification

The majority of the peptide migration was due to 18 identified and semi-quantified sequences (Table 1). These sequences represent 79.84 ± 0.94% of the total area under the curve of the peptide recovery fraction.

**Figure 4:**
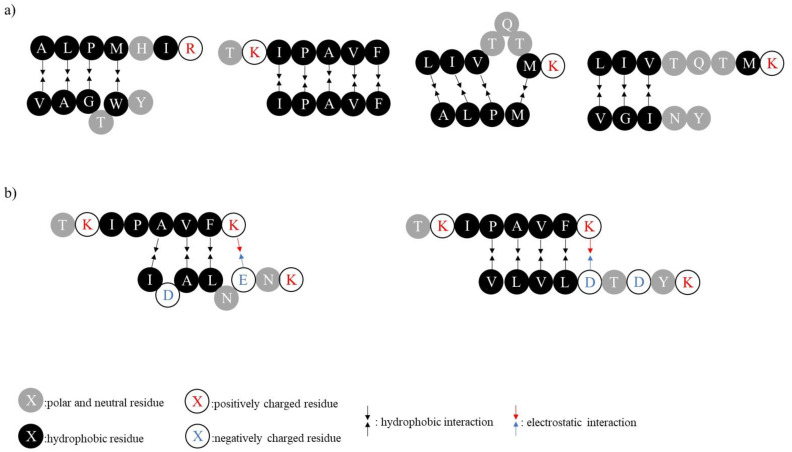
Potential interactions between positively charged peptides and (**a**) neutral peptides or (**b**) negatively charged peptides to form globally positively charged aggregates that can migrate during EDUF treatment.

Negatively charged peptides, neutral peptides and positively charged peptides were recovered in the peptide recovery fraction. Such a phenomenon was already reported in the literature for the specific recovery of cationic peptides with different scales of EDUF cells [6,10,14,15]. However, surprisingly, the majority of the recovered peptides were neutral peptides, while in previous works, only a few neutral peptides were found. Furthermore, the relative abundance of certain neutral peptides, such as VAGTWY, IPAVF and YLLF, increased in the peptide recovery compartment compared to the initial WPH. During EDUF treatment, neutral peptides are theoretically not supposed to migrate, as they are not charged. However, as previously suggested, it could be possible for them to form positively charged aggregates with positively charged peptides via electrostatic and/or hydrophobic interactions. These aggregates could migrate from the WPH to the peptide recovery fraction. Indeed, the positively charged peptides found in the peptide recovery fraction present hydrophobic residues, or even the hydrophilic tails of several residues (such as ALPMHIR, TKIPAVK and LIVTQTMK), just like the neutral peptides recovered. Thus, Figure 4a presents examples of globally charged aggregates that could be formed between the positively charged peptides and few neutral peptides via mostly hydrophobic interactions. Concerning the negatively charged peptides IDALNENK and VLVLDTDYK, an impoverishment occurred during EDUF treatment, as their relative abundance in the peptide recovery fraction represents nearly 0.31 X and 0.40 X their relative abundance in the initial WPH, respectively. However, their presence in the peptide recovery fraction could be explained by interactions between positively charged peptides to form aggregates with a global positive charge, just as for the neutral peptides. Even though peptides with several positive charges are not among the major components recovered in the peptide recovery fraction, a few were still present (e.g., TKIPAVFK) and could have interacted with negatively charged peptides via electrostatic and/or hydrophobic interactions (Figure 4b). Concerning the wanted positively charged peptides, the EDUF treatment increased the relative abundance of IVTQTMK, ALPMHIR + PMHIR, LIVTQTMK and TKIPAVF to nearly 1.23 X, 7.55 X, 1.43 X and 2.28 X, respectively, in the peptide recovery fraction compared to their abundance in the initial WPH, indicating their enrichment in the recovered fraction (Table 1).

**Figure 5:**
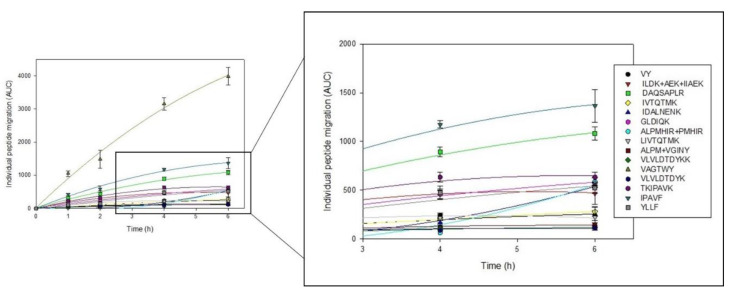
Evolution as a function of time of the individual migration of the major components found in the peptide recovery fraction (LC-UV 214 nm data).

Concerning the individual peptide migration (Figure 5), different behaviors can be observed, as the peptide migration may change during EDUF treatment. First, one can notice that the migration of VAGWTY tended to increase linearly during treatment. Furthermore, VLVLDTDYKK, VLVLDTDYK and ALPM + VGINY had a relatively constant and low migration during treatment. For ALPMHIR + PMHIR and IDALNENK, the migration was quite low, but it increased after 4 h of treatment. For most peptides, their migration increased during treatment to move towards a plateau after 4 h of treatment. Thus, depending on the peptides wanted in the peptide recovery fraction, it is possible to adjust the treatment time, as previously suggested [14]. Moreover, this can be confirmed by the different final migration rates (Table 1), which also varied according to eachpeptide.

Some factors could explain the differences in treatment selectivity from the one previously reported for EDUF separation using the same semi-industrial cell and configuration [14]. One factor that can be determinant in this phenomenon is the completely different peptide population of the initial WPH. Indeed, in the present work, the hydrolysis was carried out using a commercial enzyme in which trypsin, but also chymotrypsin, was found, while in the previous work, a pure trypsin enzyme was used. This enzyme mixture can be a source of many new peptides that could have affected the migration dynamics during EDUF treatment. Furthermore, in this work, a WPC was used (instead of a WPI), which also could have affected the initial peptide population. The other factors that could have had an impact on the selectivity of the treatment are related to the high conductivity of the initial WPH. Indeed, such a high conductivity in the WPH, even if it decreased during treatment (Figure 2a), resulted in a lower local electric field strength than the one previously reported, which could have affected peptide migration, particularly in terms of treatment selectivity [14]. Furthermore, the high conductivity led to a consequently high ionic strength in the WPH, lowering the solubility of the peptides. In this case, peptide–peptide interactions were favored, through the mechanisms previously discussed [14]. Thus, it could explain the presence of a large number of neutral peptides in the peptide recovery fraction after treatment as well as the higher peptide yield, following high ionic strength.

It is also important to mention that, as expected, the demineralization after EDUF treatment did not affect the peptide profiles and quantities of the different fractions produced. In all cases (data not shown), the chromatograms between the fractions after EDUF and after demineralization overlapped well. Thus, the peptides found in the demineralized fractions are the same as those found in the non-demineralized fraction.

### 3.4. Bioactivities

The biological activities of the different fractions produced during treatment are presented in Table 2. There are significant differences between the IC_50_ values for the inhibition of DPP-IV and ACE (*p* = 0.004 and *p* < 0.001, respectively), as the peptide recovery fraction presents lower values than the initial and final WPHs. Indeed, the process allows the production of a fraction with improved biological activities, as the IC_50_ values are reduced by nearly 2 X and 4 X, respectively, compared to the initial WPH, with a final peptide yield of only 5.46 ± 0.56% (Figure 3b). This decrease in the IC_50_ values for the inhibition of both components is due to the enrichment in the fraction of certain bioactive peptides in sufficient quantities to increase its activity. Indeed, in the case of DPP-IV, VAGTWY and IPAVF, which have demonstrated an activity in its inhibition [26], are concentrated in the peptide recovery fraction during EDUF treatment, as their relative abundance increased in this fraction compared to their relative abundance in the initial WPH (Table 1). Furthermore, the fraction is enriched in peptides, such as IIAEK and LIVTQTMK, that have structural characteristics that could inhibit DPP-IV, suggesting they could also have an inhibitory activity [27]. These peptides present specific hydrophilic residues at the N-terminus that could interact with the hydrophilic pocket of the active site of DPP-IV, limiting its activity [28,29]. Thus, the presence of these peptides in the peptide recovery fraction could also enhance its inhibitory activity. As previously demonstrated on another WPH, the process allows the production, in relatively large quantities, of a fraction with a DPP-IV inhibitory activity similar to fractions produced using chromatographic techniques [14]. For ACE, different peptides with individual low IC_50_ values, such as IIAEK, ALPMHIR and YLLF, were recovered and concentrated in the peptide recovery fraction, along with other peptides with a lower ACE inhibitory activity, such as GLDIQK, ALPM and VAGTWY (Table 1) [26,30,31,32,33]. The presence of these peptides in the peptide recovery fraction could explain its promising activity to inhibit ACE.

For the antioxidant activity of the fractions, there is no significant difference between them (*p* = 0.449) (Table 2). During EDUF treatment, VAGTWY, an antioxidant peptide [26], was concentrated in the peptide recovery fraction. However, it is possible that the treatment did not allow it to be concentrated in sufficient concentration to increase the antioxidant activity of the fraction. Furthermore, it is also possible that the bioactive peptides found in the fractions present a different antioxidant mechanism than the one tested here, leading to no difference between the fractions.

### 3.5. Lactose Transfer

Interestingly, lactose was also recovered in the peptide recovery fraction in addition to the migration of peptides. Effectively, during EDUF treatment, 29.75 ± 1.20 g of lactose was transferred from the hydrolysate to the recovery fraction, corresponding to 25.61 ± 1.03% of total lactose found in the initial WPH. It is the first time that such a phenomenon has been reported during EDUF treatment with a WPH. Indeed, in this work, a WPC, which has a content of 41.29 ± 1.86 g/100 g of WPC35 on a dry basis, was used to prepare the WPH. In previous works [14], WPHs were prepared, rather, with a WPI, a product in which lactose content is residual [34]. This could explain why this phenomenon has not been observed before.

Nevertheless, a hypothesis that can be put forward to explain the recorded lactose transfer where lactose would pass through the UF membrane by diffusion. This phenomenon would be possible, even if no pressure was applied, since lactose has a size of 0.8 nm and UF membrane pores, rather, have a size of 2–100 nm [35]. Furthermore, according to manufacturer’s data, the type of UF membrane used has a very low lactose retention coefficient, which could also support the highlighted hypothesis concerning the diffusion phenomenon.

## 4. Conclusions

Previously, promising results were obtained for peptide separation by EDUF at a semi-industrial scale. In order to continue the development of this technology at a larger scale, a new hydrolysate, generated from a common dairy product (WPC35), was separated to recover bioactive peptides. This new hydrolysate led to high peptide migration and recovery throughout treatment. Furthermore, the relative abundance of positively charged peptides increased in the peptide recovery fraction compared to the initial hydrolysate. Nevertheless, many neutral peptides were recovered along with them, thus affecting the selectivity of the process. The selectivity observed could result from the peptide population obtained with the enzyme mixture (trypsin and chymotrypsin) and the product (WPC35) used to carry out the hydrolysis. Nevertheless, peptide–peptide interactions due to the high initial conductivity of the WPH could have occurred, favoring neutral peptide migration by interaction (hydrophobic or electrostatic) with positively-charged peptides. Despite that, the peptide recovery fraction that was produced is promising for its potential health effects, as it had an improved *in vitro* activity for the inhibition of DPP-IV and ACE, suggesting antidiabetic and antihypertensive activities. In addition, *in vivo* assays are currently underway with the produced demineralized fractions to assess their activity on the physiological functions associated with metabolic syndrome combined with obesity and on the prevention of high blood pressure.

Nevertheless, in this work, it was demonstrated that a WPC, a common and accessible product from the dairy industry, could be used as a source of bioactive peptides and could result in a product with a high added value. Thus, it comes very close to what could be achieved on a large scale to produce a fraction that contains bioactive peptides from whey proteins. However, an avenue to consider for improving process selectivity, while remaining under conditions close to industrial scale, would be to use a WPC55 instead of a WPC35 to produce the initial WPH. Since this product contains less minerals and lactose than a WPC35, the peptide recovery fraction produced may be further enriched with positively charged peptides, as the lower ionic strength could limit neutral peptide migration, while possibly having less lactose.

## Figures and Tables

**Table 1:** Major components recovered in the peptide recovery fraction: characteristics, relative abundance (in%), concentration factor (in X) and final migration rate (in X).

Peptide Sequence	Observed Mass (Da)	Retention Time (min)	Net Charge at pH7	Peptide Source	Relative Abundance in Peptide Recovery Fraction *	Relative Abundance in Initial WPH **	Concentration factor in the Peptide Recovery Fraction	Final Migration Rate
VY	280.14	2.5	0	b-lg (41–42)	3.75 ± 1.25	2.43 ± 0.48	1.53 ± 0.37	22.78 ± 2.24
ILDK + AEK+IIAEK	487.30, 346.15, 572.35	2.8	0, 0, 0	a-la (95–98), b-lg (73–75), b-lg (71–75)	4.95 ± 0.41	4.52 ± 0.80	1.12 ± 0.24	81.55 ± 2.47
DAQSAPLR	856.44	4.04	0	b-lg (33–40)	3.53 ± 0.26	3.43 ± 0.19	1.03 ± 0.09	52.13 ± 10.05
IVTQTMK	819.45	4.4	1	b-lg (2–8)	1.41 ± 0.11	1.14 ± 0.06	1.25 ± 0.14	27.60 ± 5.98
IDALNENK	915.47	5.1	−1	b-lg (84–91)	0.45 ± 0.00	1.43 ± 0.03	0.31 ± 0.01	72.32 ± 16.94
GLDIQK	672.38	5.6	0	b-lg (9–14)	3.08 ± 0.06	3.50 ± 0.04	0.88 ± 0.01	37.06 ± 8.31
ALPMHIR + PMHIR	836.47, 656.35	7.91	1.1, 1.1	b-lg (142–148), b-lg (144–148)	1.66 ± 0.14	0.22 ± 0.00	7.52 ± 0.63	24.34 ± 1.84
LIVTQTMK	932.54	8.26	1	b-lg (1–8)	5.55 ± 0.28	3.88 ± 0.11	1.43 ± 0.03	8.86 ± 2.84
ALPM + VGINY	430.22, 564.29	8.63	0, 0	b-lg (142–145), a-la (99–103)	5.41 ± 0.08	3.33 ± 0.42	1.64 ± 0.24	3.84 ± 0.64
VLVLDTDYKK	1192.67	9.32	0	b-lg (92–101)	0.80 ± 0.05	3.14 ± 0.53	0.23 ± 0.03	14.68 ± 0.88
VAGTWY	695.33	10.62	0	b-lg (15–20)	28.04 ± 0.65	13.26 ± 0.35	2.11 ± 0.01	18.69 ± 1.19
VLVLDTDYK	1064.57	11.25	-1	b-lg (92–100)	1.02 ± 0.17	2.55 ± 0.31	0.40 ± 0.05	3.20 ± 0.52
TKIPAVF	774.46	12.27	1	b-lg (76–82)	5.94 ± 0.36	2.61 ± 0.23	2.28 ± 0.08	66.01 ± 4.76

b-lg: b-lactoglobulin, a-la: a-lactalbumin, WPH: whey protein hydrolysate; * values calculated from AUC (LC–UV data) for the peptide recovery fraction after 6 h of treatment, total of the major components is 79.87 ± 0.94%; ** values calculated from AUC (LC–UV data) for the initial whey protein hydrolysate, total of the major components is 51.04 ± 1.33%.

**Table 2:** DPP-IV half-maximal inhibitory concentration (in mg/mL), ACE half-maximal inhibitory concentration (in mg/mL) and ORAC values (in mmol TE/g peptide) of the demineralized fractions.

	IC_50_ DPP-IV Inhibition	IC_50_ ACE Inhibition	ORAC
Demineralized initial WPH	0.9734 ± 0.0463 ^a^	44.11 ± 0.43 ^a^	716.2 ± 96.8 ^a^
Demineralized final WPH	1.2175 ± 0.3781 ^a^	22.96 ± 0.58 ^b^	804.2 ± 234.3 ^a^
Demineralized peptide recovery fraction	0.5029 ± 0.0796 ^b^	10.62 ± 1.13 ^c^	824.8 ± 80.6 ^a^

DPP-IV: dipeptidyl-peptidase, IC_50_: half-maximal inhibitory concentration, ACE: angiotensin-converting enzyme, ORAC: oxygen radical absorbance capacity, TE: Trolox equivalent, WPH: whey protein hydrolysate. Column-wise: values with different letters are significantly different, *p* < 0.05 (Tukey test).

## Data Availability

Not applicable.

## References

[1] Dullius A., Goettert M.I., de Souza C.F.V. (2018). Whey Protein Hydrolysates as a Source of Bioactive Peptides for Functional Foods—Biotechnological Facilitation of Industrial Scale-Up. J. Funct. Foods.

[2] Pouliot Y., Wijers M.C., Gauthier S.F., Nadeau L. (1999). Fractionation of Whey Protein Hydrolysates Using Charged UF/NF Membranes. J. Membr. Sci..

[3] Hannu K., Pihlanto A. (2006). Bioactive Peptides: Production and Functionality. Int. Dairy J..

[4] Dlask O., Vaclavikova N. (2018). Electrodialysis with Ultrafiltration Membranes for Peptide Separation. Chem. Pap..

[5] Sun L., Chen Q., Lu H., Wang J., Zhao J., Li P. (2020). Electrodialysis with Porous Membrane for Bioproduct Separation: Technology, Features, and Progress. Food Res. Int..

[6] Bazinet L., Geoffroy T.R. (2020). Electrodialytic Processes: Market Overview, Membrane Phenomena, Recent Developments and Sustainable Strategies. Membranes.

[7] Durand R., Fraboulet E., Marette A., Bazinet L. (2019). Separation and Purification Technology Simultaneous Double Cationic and Anionic Molecule Separation from Herring Milt Hydrolysate and Impact on Resulting Fraction Bioactivities. Sep. Purif. Technol..

[8] Przybylski R., Bazinet L., Firdaous L., Kouach M., Goossens J.-F., Dhulster P., Nedjar N. (2020). Harnessing Slaughterhouse By-Products: From Wastes to High-Added Value Natural Food Preservative. Food Chem..

[9] He R., Girgih A.T., Rozoy E., Bazinet L., Ju X., Aluko R.E. (2016). Selective Separation and Concentration of Antihypertensive Peptides from Rapeseed Protein Hydrolysate by Electrodialysis with Ultrafiltration Membranes. Food Chem..

[10] Kadel S., Daigle G., Thibodeau J., Perreault V., Pellerin G., Lainé C., Bazinet L. (2021). How Physicochemical Properties of Filtration Membranes Impact Peptide Migration and Selectivity during Electrodialysis with Filtration Membranes: Development of Predictive Statistical Models and Understanding of Mechanisms Involved. J. Membr. Sci..

[11] Suwal S., Roblet C., Doyen A., Amiot J., Beaulieu L., Legault J., Bazinet L. (2014). Electrodialytic Separation of Peptides from Snow Crab By-Product Hydrolysate: Effect of Cell Configuration on Peptide Selectivity and Local Electric Field. Sep. Purif. Technol..

[12] Poulin J.-F., Amiot J., Bazinet L. (2008). Impact of Feed Solution Flow Rate on Peptide Fractionation by Electrodialysis with Ultrafiltration Membrane. J. Agric. Food Chem..

[13] Roblet C., Doyen A., Amiot J., Bazinet L. (2013). Impact of PH on Ultrafiltration Membrane Selectivity during Electrodialysis with Ultrafiltration Membrane (EDUF) Purification of Soy Peptides from a Complex Matrix. J. Membr. Sci..

[14] Geoffroy T.R., Bernier M.E., Thibodeau J., Francezon N., Beaulieu L., Mikhaylin S., Langevin M.E., Lutin F., Bazinet L. (2022). Semi-Industrial Scale-up of EDUF Technology for the Electroseparation of Bioactive Cationic Peptides: Impact of Process Parameters and Cell. J. Membr. Sci..

[15] Geoffroy T.R., Thibodeau J., Faucher M., Langevin M.È., Lutin F., Bazinet L. (2022). Relationship between Feed Concentration and Biactive Cationic Peptide Recovery: Impact on Ecoefficiency of EDUF at Semi-Industrial Scale. Sep. Purif. Technol..

[16] Kadel S., Pellerin G., Thibodeau J., Perreault V., Lainé C., Bazinet L. (2019). How Molecular Weight Cut-Offs and Physicochemical Properties of Polyether Sulfone Membranes Affect Peptide Migration and Selectivity during Electrodialysis with Filtration Membranes. Membranes.

[17] Galier S., Balmann H.R. (2004). Study of Biomolecules Separation in an Electrophoretic Membrane Contactor. J. Membr. Sci..

[18] Maubois J.-L., Lorient D. (2016). Dairy Proteins and Soy Proteins in Infant Foods Nitrogen-to-Protein Conversion Factors. Dairy Sci. Technol..

[19] Silveira S.T., Martínez-maqueda D., Recio I., Hernández-ledesma B. (2013). Dipeptidyl Peptidase-IV Inhibitory Peptides Generated by Tryptic Hydrolysis of a Whey Protein Concentrate Rich in b-Lactoglobulin. Food Chem..

[20] Hayakari M., Kondo Y., Izumi H. (1978). And Simple Spectrophotometric Assay of Enzyme. Anal. Biochem..

[21] Hell A., Labrie S., Beaulieu L. (2018). Effect of Seaweed Flakes Addition on the Development of Bioactivities in Functional Camembert-Type Cheese. Int. J. Food Sci. Technol..

[22] Held P. (2006). Performing Oxygen Radical Absorbance Capacity Assays with SynergyTM HT.

[23] (2007). Milk and Milk Products-Determining of Lactose Content by High-Performance Liquid Chromatography (Reference Method).

[24] Persico M., Dhulster P., Bazinet L. (2018). Redundancy Analysis for Determination of the Main Physicochemical Characteristics of Filtration Membranes Explaining Their Fouling by Peptides. J. Membr. Sci..

[25] Persico M., Daigle G., Kadel S., Perreault V., Pellerin G. (2020). Predictive Models for Determination of Peptide Fouling Based on the Physicochemical Characteristics of Filtration Membranes. Sep. Purif. Technol..

[26] Power O., Fernández A., Norris R., Riera F.A., Fitzgerald R.J. (2014). Selective Enrichment of Bioactive Properties during Ultrafiltration of a Tryptic Digest of β-Lactoglobulin. J. Funct. Foods.

[27] Nongonierma A.B., Mazzocchi C., Paolella S., Fitzgerald R.J. (2017). Release of Dipeptidyl Peptidase IV (DPP-IV) Inhibitory Peptides from Milk Protein Isolate (MPI) during Enzymatic Hydrolysis. Food Res. Int..

[28] Engel M., Hoffmann T., Wagner L., Wermann M., Heiser U., Kiefersauer R., Huber R., Bode W., Demuth H., Brandstetter H. (2003). The Crystal Structure of Dipeptidyl Peptidase IV (CD26) Reveals Its Functional Regulation and Enzymatic Mechanism. Proc. Natl. Acad. Sci. USA.

[29] Nongonierma A.B., Fitzgerald R.J. (2014). Susceptibility of Milk Protein-Derived Peptides to Dipeptidyl Peptidase IV (DPP-IV) Hydrolysis. Food Chem..

[30] Murakami M., Tonouchi H., Takahashi R.R., Kitazawa H., Kawai Y., Negishi H., Saito T. (2004). Structural Analysis of a New Antihypertensive Peptide (β-Lactosin B) Isolated from a Commercial Whey Product. J. Dairy Sci..

[31] Pihlanto-Leppälä A., Rokka T., Korhonen H. (1998). Angiotensin I Converting Enzyme Inhibitory Peptides Derived from Bovine Milk Proteins. Int. Dairy J..

[32] Pihlanto-Leppälä A., Koskinen P., Piilola K., Tupasela T., Korhonen H. (2000). Angiotensin I-Converting Enzyme Inhibitory Properties of Whey Protein Digests: Concentration and Characterization of Active Peptides. J. Dairy Res..

[33] Mullaly M.M., Meisel H., Fitzgerald R.J. (1996). Synthetic Peptides Corresponding to Alpha-Lactalbumin and Beta-Lactoglobulin Sequences with Angiotensin-I-Converting Enzyme Inhibitory Activity. Biol. Chem. Hoppe. Seyler..

[34] De Boer R. (2014). Information Sheets. From Milk By-Products to Milk Ingredients: Upgrading the Cycle.

[35] De Boer R. (2014). Vital Membrane Processes. From Milk By-Products to Milk Ingredients: Upgrading the Cycle.

